# Efficient Degradation of Industrial Biowaste via In-Vessel Composting—Technical and Microbial Assessments

**DOI:** 10.3390/bioengineering12010033

**Published:** 2025-01-04

**Authors:** Jamie Jean Minn Tan, Zi Xiang Keng, Siew Hui Chong, Guan-Ting Pan, Ajit Singh, Christinavimala Supramaniam, Ianatul Khoiroh

**Affiliations:** 1 School of Biosciences, University of Nottingham, Broga Road, Semenyih 43500, Selangor, Malaysia; jtjay14@gmail.com (J.J.M.T.); ajit.singh@nottingham.edu.my (A.S.); 2 Department of Chemical and Environmental Engineering, University of Nottingham, Broga Road, Semenyih 43500, Selangor, Malaysia; jaronkeng@gmail.com (Z.X.K.); faye.chong@xodusgroup.com (S.H.C.); 3 Xodus Group, Level 1/1 William Street, Perth, WA 6000, Australia; 4 College of Science, Health, Engineering and Education, Murdoch University, 90 South Street, Murdoch, WA 6150, Australia; kuan-ting.pan@murdoch.edu.au; 5 School of Science, The University of Greenwich, Chatham ME4 4TB, UK

**Keywords:** in-vessel composting, metagenomic, bacterial, fungi, biowaste, compost

## Abstract

In this study, a pilot-scale in-vessel composter was used to treat a mixture of industrial biowaste, with soybean curd residue and saw dust as the major substrates. The composter is capable of treating up to 350 tons/month of waste, producing up to 150 tons/month of high-quality compost within a retention time of 7–10 days. The final compost has an average nitrogen–phosphorus–potassium content of 6%, moisture content of 28%, pH of 6.1, organic matter of 68%, and carbon–nitrogen ratio of 19:1. It also has a good amount of humic acid and macronutrients. Composts from all stages of the composting process—pre-mix, directly after discharge, after one-month of curing, and right before packaging—were evaluated with metagenomic analysis to identify the microbes that may add value to the compost.

## 1. Introduction

Commercial composting supplies the agricultural industry with a nutrient-rich compost for soil enrichment as an alternative to chemical fertilizers, with composting referring to the bioconversion of organic matter into the stabilized end-product [1,2]. Consisting of two degradation stages, the primary composting stage—also known as the mesophilic stage—involves the metabolism of the easily degradable organic substrates, leading to an increase in temperature to a thermophilic range. The stage where the compost has a high temperature is the thermophilic stage. Once the temperature drops back down from the thermophilic range to near an ambient temperature, the secondary composting stage or maturation takes place with the degradation of the organic substrates that had not been metabolized in the primary stage [3,4]. 

This degradation process is dictated by microorganisms, with bacteria and fungi playing notable roles. Bacteria with their high metabolic versatility, and fungi with their ability to survive different conditions such as dry and acidic conditions and to utilize many different carbon substrates, allow for the breakdown of most organic compounds when composting, resulting in a humus- and nutrient-rich end product [1,4]. The microbial abundance and diversity of the compost determine its quality. When certain microbes with different enzymatic properties are present, the compost is of higher quality and can gain added-value characteristics such as resistance against phytopathogens through suppressive activity [4]. In a similar vein, the lack of sufficient microbial activity leads to a compost of poor quality as a result of incomplete breakdown of organic substrates [5]. Thus, studies have previously assessed the microbial composition of composts to understand the changes it undergoes during the different composting stages [6,7]. This determination of microbial abundance and diversity was achieved by metagenomic analysis.

Metagenomics refers to the study of the microbial composition in environmental samples by means of direct genetic analysis of microbial genomes. In metagenomic analysis, DNA is extracted from the culture-independent sample and the major marker genes (16S rRNA) for prokaryotes and internal transcribed spacer (ITS) are targeted for amplification, constructing a library of amplicons that is then analyzed to produce genomic data of the bacterial and fungal composition in the sample [8]. The data produced from the genomic analysis can then be utilized in the taxonomic determination of the microbes present and the measurement of the sample’s microbial diversity, thus allowing for the study of the overall function of the microbial community as well as phylotyping and exploration of complex microbial interactions [9]. The metagenomic approach uses culture-independent samples taken directly from the environment of interest as opposed to pure culture samples, as the controlled parameters of a pure culture is unable to truly represent the various environmental conditions that affect the microbial composition in a sample [9]. This approach has been successfully applied in previous compost studies exploring the link between the microbial composition in the compost sample and its function in the composting process, as well as the factors that lead to compositional changes in the microbes present [7,10]. In a study by Antunes et al. [7] that utilized the metagenomic approach, aeration, nutrient access, and temperature were found to affect the microbial composition in a compost sample. As such, the ability to profile and perform taxonomic assignment for an environmental sample as well as its production of a genomic dataset for analysis led to the decision to use the metagenomic analysis method for this study.

The method of composting is another variable that affects compost quality. Conventional open-air composting (OAC) involves methods such as the aerated static pile method, open air windrow, deposited composting, and tunnel agitated composting methods [11,12]. However, the OAC method requires a long composting duration of 4–5 months and takes up a large amount of space, and even so, it produces a still active end-product of acidic (pH 4–5) and high-moisture (~50%) compost. An alternative composting method to OAC is in-vessel composting (IVC). IVC systems can be loosely defined as structures built purposefully to house the composting process [13]. The system can be designed to be anaerobic—for anaerobic digestion of the organic material, instead of composting—or aerobic, but Iyengar and Bhave [14] assessed the efficiency of household compost reactor designs to compost household biowastes and found that an aerobic IVC design was more effective in producing nutrient-rich compost for soil enrichment. As such, most IVC composter designs aim to provide a more effective aerobic composting process with negligible odor emission and a shorter composting duration to reach a similar level of end-compost maturity, as well as the ability to produce compost at a greater maturity and occupy less space while processing at a similar capacity. These characteristics are encouraging a shift towards IVC use however, the capital investment, operation expenditure, and operation complexity of IVC are notably much higher than those of OAC.

The initial feedstock also largely influences the microbial composition and characteristics of the compost as they affect the pH of the resultant compost and provide the carbon and nitrogen sources for microbial growth and metabolism [2]. Pendey et al. [15] used a feedstock mixture of food waste, horse manure, and palm leaves in their designed IVC system, while Hernandez-Lara et al. [4] studied the effect of different agro-industry wastes as feedstock on the microbial communities in the compost, finding significant differences in abundance and diversity between the piles. Various buy-back substrates and wastes from the agricultural industry can be potential compost feedstocks and the choice of substrates can affect the end-product quality.

In this study, a pilot-scale in-vessel composter was utilized, and the compost produced from a mixture of agricultural waste—soybean curd residues (SCRs) and sawdust—was sampled and analyzed. The objectives of this research were to (1) assess the effectiveness (design capacity) of a large-scale, aerobic, IVC composter design to produce quality fertilizer (NPK%, C/N ratio, moisture, etc.) with commercial potential, (2) analyze the bacterial and fungal composition of compost samples produced from buy-back feedstocks of soybean curd residues and sawdust through metagenomic analysis, and (3) identify microbes that contribute to the composting process and add value to the compost quality.

## 2. Methodology

### 2.1. IVC System Design

In this work, the IVC system [dataset] is proprietary to a third party. The entire operation mechanism was designed to be a vertical process with air input from the bottom for moisture control. The system was modified to be without an external heat source. Instead, a thermal insulator was included in the structural design to maintain the composting heat by insulation and prevention of heat loss. The material used for this was a reliable three-layered heat insulating material that conformed to all environments, hence its use as the system’s wall material. Mechanical turning was performed by propellers with a 15 min run, 15 stop cycle, or about 30 min of turning per hour at 8–13 MPa. To control odor, a deodorization system with an exhaust was included in the design. The whole IVC system was designed to be leak-proof and to allow for the regular adding of microbes during the infeed of pre-mixed substrates.

### 2.2. Composting Procedure

**Figure 1:**
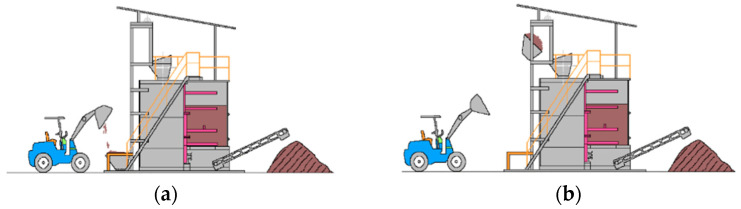

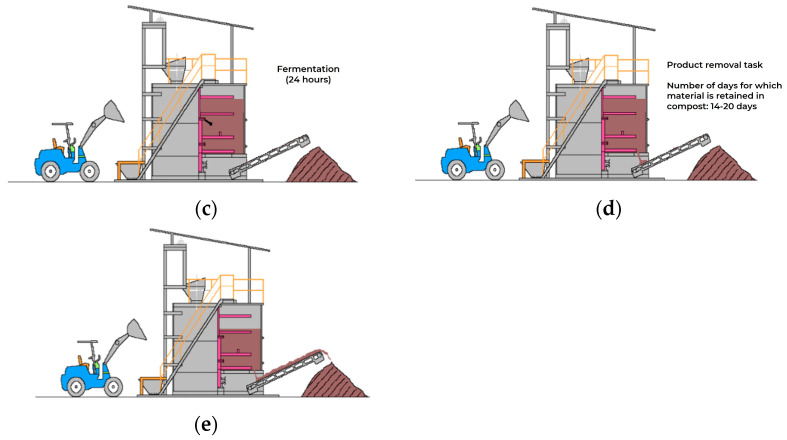
(**a**) Step 1—Pre-mixed substrates are fed into the loading bucket; (**b**) Step 2—The feedstock is lifted by the bucket elevator and loaded into the IVC composter; (**c**) Step 3—Fermentation takes place; (**d**) Step 4—Substrates are retained and fermented in-tank for a duration of 14–20 days; and (**e**) Step 5—Output release of organic fertilizer.

Figure 1 illustrates the composting procedure. The pre-mixed partially fermented material (SCR and sawdust) is fed into the IVC system using a loading bucket with a volume of 1.1 m^3^. From the loading bucket, the pre-mixed materials are deposited into the feeding bucket, which is then lifted by the bucket elevator for loading into the IVC composter. The process begins and infeed materials are composted inside the IVC tank with agitation for 24 h and continuous injection of air. Compost is discharged daily, making room for the infeed of feedstock daily. The in-tank retention time of infeed materials is about 14–20 days, while the operation of the IVC is a continuous process with feeding and discharge occurring once or a maximum of twice per day.

### 2.3. Compost Quality and Characterization of Feedstock and Finished Compost

The basic characteristics and nutrient values were assessed through laboratory tests while the stability and maturity level of compost output were assessed using the Solvita test. The laboratory tests were conducted on three separate occasions (September 2020, October 2020, and January 2021) for accuracy. Table 1 summarizes the characterization methods used in this study for the feedstock and finished compost.

### 2.4. Calculations for Carbon–Nitrogen Ratio, Moisture, and Compost Quantity

To achieve the desired initial carbon–nitrogen ratio (*C*:*N*) and moisture content (*W*) of 25~35 and 55~70%, respectively, the amounts of substrates were optimized using Microsoft Excel Solver based on Equations (1) and (2) [16]:

(1) $$C\text{:}N=\frac{M_{1}\left[C_{1}\times \left(100-W_{1}\right)\right]+M_{2}\left[C_{2}\times \left(100-W_{2}\right)\right]+M_{3}\left[C_{3}\times \left(100-W_{3}\right)\right]+\ldots }{M_{1}\left[N_{1}\times \left(100-W_{1}\right)\right]+M_{2}\left[N_{2}\times \left(100-W_{2}\right)\right]+M_{3}\left[N_{3}\times \left(100-W_{3}\right)\right]+\ldots },$$

(2) $$W(\%)=\frac{M_{1}W_{1}+M_{2}W_{2}+M_{3}W_{3}+\ldots }{M_{1}+M_{2}+M_{3}+\ldots }$$

where *M*: weight of substrates (%), *C*: carbon content of substrates (%), *W*: moisture content of substrates (%), and *N*: nitrogen content of substrates (%) with the subscript indicating the substrate number. In this project, the total organic carbon and total organic nitrogen, respectively, represented the *C* and *N* utilized.

A series of calculations, Equations (3)–(5), rearranged from the IVC manufacturer’s manual were conducted to calculate the amount of output compost produced.

Amount of water evaporated by fermentation heat, *E*_1_ in kg/d:

(3) $$E_{1}=\frac{0.025t}{k_{1}}\;\sum Q_{i}{CV}_{i}\;(1-W_{i})$$

where 0.025*t* is the decomposition rate with *t* as the lead time or retention time, in days; the subscript i indicates each substrate while *Q* and *CV* are the amount (kg/d) and calorific value of substrate (kcal), respectively; *W* refers to the moisture content as a fraction and the constant *k*_1_ is the quantity of heat per 1 kg of water evaporation, equivalent to 700 kcal (Manual, SKS-9610).

Water amount of evaporation by warm air ventilation, *E*_2_ in kg/d:

(4) $$E_{2}=k_{2}\;(\frac{t}{2}\times q_{a}\;)(\frac{\Sigma Q_{i}}{Z}+Q_{c}/\rho_{c})$$

where *k*_2_ is the constant accounting for the water evaporated by warm air, which equals 0.03 kg/m^3^; *q_a_* is the ventilation rate at 432 m^3^/d; Z is the maximum capacity of the composter at 700 kg/m^3^; *Q_c_* is the compost quantity produced in kg/d; ρ*_c_* is the compost bulk density, which is 400 kg/m^3^; and Equation (4) is expressed in terms of *Q_c_*.

Lastly, Equation (5) relates the amount of compost produced from IVC with the water evaporation and decomposition rate:

(5) $$Q_{c}=\left(1-0.025t\right)\Sigma Q_{i}\left(1-W_{i}\right)+\Sigma Q_{i}W_{i}-E_{1}-E_{2}$$

Equations (4) and (5) are solved simultaneously to determine the compost quantity, $Q_{c}$.

### 2.5. Design Capacity

The in-vessel composter design capacity was based on the characteristics and quantities of input materials (aka substrates) and a series of design factors (e.g., decomposition rate per day and processing duration). Three scenarios were proposed with different formulations of substrates:

- Scenario A: Other [dataset], SCR (dry), SCR (wet), sawdust, and partially fermented compost.
- Scenario B: SCR (dry), SCR (wet), and sawdust.
- Scenario C: Sawdust and partially fermented compost.

where partially fermented compost refers to the compost from open-air composting (OAC) of SCR and sawdust mixture for 1–2 weeks.

The resultant output from IVC of the three substrate scenarios was calculated using Equations (3)–(5). The amount of compost produced (tons/month), moisture content (%), and lead time taken to produce compost (days) were considered when selecting the most optimal substrate scenario for input capacity. The selected substrate formulation would then be used in IVC to produce the compost that is later sampled for metagenomic analysis.

### 2.6. Methodology of Analysis of Bacterial and Fungal Composition of IVC Compost

#### 2.6.1. Random Sampling for Metagenomic Analysis

Random sampling was performed with the collection of a sample of 50 g from five different points from the compost discharged from the in-vessel composter at different stages:

- Stage I, which contains only the pre-mixed infeed materials;
- Stage II, which is the compost directly after discharge from the IVC;
- Stage III, which is the compost from the IVC after a month of post-treatment;
- Stage IV, the compost right before packaging.

The random samples were transported in a cold storage box to the laboratory, where the 5 random sample points of each stage were mixed together thoroughly to produce a composite sample for each IVC stage.

Next, from the total 4 composite samples—one for each stage—5 assays were prepared for further DNA extraction with one bacterial assay per each stage I–IV and an additional fungal assay prepared from the composite sample of stage IV. Thus, 4 bacterial assays and 1 fungal assay were prepared for further DNA extraction with different primers.

The random sampling steps were repeated in the two months following the first sampling to produce biological replicates. Therefore, a total of 15 samples with 5 samples per random sampling conducted were produced.

#### 2.6.2. DNA Extraction and Verification

The 15 samples—3 replicates each of samples from Stages I to IV with 3 replicates of additional samples from Stage IV for fungi—were stored at 4 °C. We collected 250 mg of each sample, and DNA extraction was performed using a DNeasy PowerSoil Pro DNA Extraction kit (QIAGEN, San Diego, CA, USA) following the manufacturer’s instructions. The extracted DNA quality and quantity were assessed using the Nanodrop technique, and samples containing a minimum volume of 20 ng/µL and 260 nm/280 nm ratio of 1.8 were selected for PCR screening. Universal primers for bacterial 16S rRNA [338F (5′-GTA CTC CTA CGG GAG GCA GCA G-3′), 533R (5′-TTA CCG CGG CTG CTG GCA C-3′)] [17] were utilized for PCR of bacterial assays Stage I–IV while universal primers for the ITS region in fungi [ITS 1f (5′-CTT GGT CAT TTA GAG GAA GTA A-3′), ITS 2 (5′-GCT GCG TTC TTC ATC GAT GC-3′)] [18] were used for the PCR of the fungal assay of Stage IV. Gel electrophoresis was performed, separating PCR products for identification of DNA samples with amplified bacterial and fungal DNA fragments. This verified the presence of bacterial and fungal DNA within the sample.

Samples with successfully amplified 16S rRNA for bacterial assays and ITS regions for fungal assays were sent to GeneSEQ Sdn Bhd (Rawang, Malaysia) for DNA sequencing by Next-Generation Sequencing (NGS) using the Illumina method. Amplicon sequencing was performed and bacterial and fungal profile analyses of samples were produced.

#### 2.6.3. Metagenomic Analysis of Bacterial Diversity of Samples via Amplicon Sequencing

For bacterial profiling, PCR was performed using WizBio HotStart PCR mastermix (WizBio, Sungnam, Korea) with the use of 341F (CCTACGGGNGGCWGCAG) and 518R (ATTACCGCGGCTGCTGG) primers to amplify the bacterial 16S V3 hypervariable region from the sample genomic DNA template. Additionally, the 5′ end of primers were incorporated with an additional 5 bases of inline barcoding. This enabled inline barcoding and different samples were amplified with different combinations of the forward and reverse inline primers.

The PCR profile utilized in this process involved running at 95 °C for 3 min followed by 28 cycles of 95 °C for 30 s, 55 °C for 20 s, and 72 °C for 20 s. The amplicon size was then visualized on gel before being normalized and pooled based on their intensity. The pooled amplicons were subsequently purified by selectively immobilizing nucleic acids onto SPRI beads, which were added into the mixture at a 1X volume. Next, the Illumina adapter sequence and dual-index DNA barcodes were incorporated into purified pooled amplicon sequences using the NEB Ultra Library preparation kit (Bio-Rad Laboratories, Inc., Hercules, CA, USA). The constructed library was quantified using a Denovix high-sensitivity assay and sequenced on an iSeq100 (Illumina, San Diego) for 2 × 150 paired-end sequencing.

To analyze the data produced by Illumina sequencing, the paired-end reads produced were overlapped using fastp v0.21, and primer removal and demultiplexing were then performed on the merged reads using cutadapt v1.18. Denoising of demultiplex trimmed reads was achieved with dada2 within the QIIME2 v2021.4 pipeline. Next, the q2-feature-classifier plugin v2024.10.0 based on the latest GTDB release r202 16S rRNA database was used to perform the taxonomic assignment of the ASVs, with ASVs assigned at least to the phylum level selected for subsequent analysis. ASV and taxonomic classification tables were then exported using QIIME2 v2021.4 tools into tab-separated values (tsv format) before being manually formatted to generate a MicrobiomeAnalyst-compatible input. Finally, the alpha and beta diversities of the constructed amplicon library were calculated using QIIME2 plug-ins.

#### 2.6.4. Metagenomic Analysis of Fungal Biodiversity of Samples via Amplicon Sequencing

For fungal profiling, a Nanopore library was first prepared and sequenced with the microbial full-length 16S rRNA sequence of samples amplified by PCR using ITS9ngs and ITS4 primers with Nanopore partial adapters on the 5′ end of primers. This was achieved by carrying out PCR using WizBio Hotstart 2X Mastermix (WizBio, Seongnam, Korea) and under the conditions of 95 °C for 3 min followed by 35 cycles of 95 °C for 20 s, 50 °C for 20 s, and 72 °C for 120 s. The amplicons produced were visualized on gel and purified by immobilization onto SPRI beads. Index PCR was then performed using the EXP-PBC001 kit (Oxford Nanopore, Oxford, UK) to produce barcoded amplicon libraries, which were pooled based on band intensity and purified using SPRI beads. Next, pooled barcoded amplicons were quantified using a Denovix high-sensitivity kit (Denovix, Wilmington, DE, USA) before 200 fmol of the amplicons was used as the input for LSK110 library preparation (Oxford Nanopore, Oxford, UK). Finally, sequencing was performed on a Nanopore Flongle Flowcell (Oxford Nanopore, Oxford, UK) for a period of 24 h.

Raw Nanopore reads underwent base-calling and demultiplexing before being filtered for only reads with both forward and reverse primer sequences. Forward/reverse primer sequences were then removed, and reads were processed with NanoClust Version 1.9. By using NanoClust, clustering, consensus generation, and abundance estimation of primer trimmed reads were performed. Thus, a count table was generated from the NanoClust output table. Chimeras were then identified and removed using uchime denovo, and the chimera-filtered count table was manually formatted for QIIME2 and/or MicrobiomeAnalyst. Next, full-length ITS1-5.8S-ITS2 and the 18SV9 gene region were extracted from consensus sequences, and non-chimeric consensus full-length ITS1-5.8S-ITS2 sequences were classified using the q2-feature-classifier trained on the UNITE database. Clusters with taxonomic assignment to at least the phylum level were selected for subsequent analysis. As such, alpha and beta diversities were calculated using QIIME2 plug-ins.

## 3. Results

### 3.1. Design Capacity and Compost Quality

Each output from the IVC of the three scenarios proposed was assessed for the amount of compost produced (tons/month), moisture content (%), and lead time taken to produce compost (days), and the resultant values are detailed in Table 2 below. These three formulations were justified based on the availability and cost of the substrates alongside the amount of compost produced from the IVC.

Scenario A resulted in a compost quantity of 149 tons/month produced in 7 days with a moisture level of 32%. However, the cost of other [dataset] is the most expensive among all the substrates, so despite having the highest output quantity, Scenario A is deemed a non-economical choice. Scenario C was chosen as the input capacity and formulation for use later in the production of compost by the IVC composter for metagenomic analysis. This decision was made due to the significantly higher output amount produced by Scenario C compared to Scenario B despite the similar input quantity. Additionally, partially fermented compost was conveniently sourced from the open-air, aerated static piles adjacent to the IVC composter on site. Hence, Scenario C was selected as the design capacity for the IVC composter: 240 tons/month of feedstock consisting of 180 tons/month of partially fermented compost and 60 tons/month of sawdust. This would yield 123 tons/month of the finished compost with a moisture level of 25% in 7 days—a 52% yield due to moisture loss and volume reduction from the degradation of organic matter.

Table 3 details the values of the key parameters assessed, while Table 4 denotes the macronutrient content of compost as assessed from the IVC output. Of the results, there were several notable values. The NPK content of the compost averaged around 6% while the moisture content was about 27% in average. The C:N ratio of IVC compost was about 19:1 on average and its conductivity averaged 5020 µS/cm. The magnesium (1.24% *w*/*w*) and calcium contents (2.21% *w*/*w*) of compost are higher than the normal range, with both being greater than 1% each.

Compost maturity refers to how complete the composting process is, with the more complete composting process having beneficial characteristics such as decreased toxicity, less odor generation, and a minimal impact on soil nitrogen [19]. The maturity of a compost sample can be assessed with various tests and indices.

One such method is the use of the Solvita test. The Solvita test of IVC compost in January 2021 achieved a maturity index of 7, albeit with a lower value in other months, which indicates that further curing is required (Table 3). A Solvita index of 6 and above indicates that the compost has reached the curing stage where aeration requirement is reduced and the compost is ready for packaging. The degree of decomposition of organic matter can also indicate the maturity of compost, with a lower amount of organic matter suggesting a more complete composting process and thus a higher degree of maturity [19]. Therefore, the amount of organic matter in the compost output from IVC composting at 60% (Table 3) indicated a relatively higher maturity level in comparison to compost outputs from OAC, which generally have a high organic matter content of ~90%.

### 3.2. Analysis of Bacterial and Fungal Composition of IVC Compost

Compost samples from Stages I, II, III and IV of the IVC method were profiled, and the bacterial and fungal ASV compositions of each stage were compared between each other in a Venn diagram.

We found 83 unique ASVs for Stage I, 16 unique ASVs for Stage II, 57 unique ASVs for Stage III, and 72 unique ASVs for Stage IV. Each of the different combinations of the four stages was shown to have shared bacterial ASVs, with 48 ASVs shared between all four stages. The top five bacterial ASVs based on OTU count that were unique to each stage can be found in Table 5 below. The top five bacterial ASVs shared among all four stages were identified to be the following:

- *Lactobacillus* (Genus);
- *Acetobacteraceae* (Family);
- *Corynebacterium* (Genus);
- *Saccharopolyspora rectivirgula* (Species);
- *Neobacillus* (Genus).

Thirty fungal clusters were found to be shared between all three replicates, suggesting that these 30 clusters are more consistently present at Stage IV of the IVC process than the other fungal clusters found. Five of the shared fungal clusters with the highest OTU counts were determined and are denoted as follows:

- *Trichosporon* (Genus);
- *Sterigmatomyces* (Genus);
- *Pichia deserticola* (Species);
- *Lichtheimia ornata* (Species);
- *Lichtheimia corymbifera* (Species).

**Figure 2:**
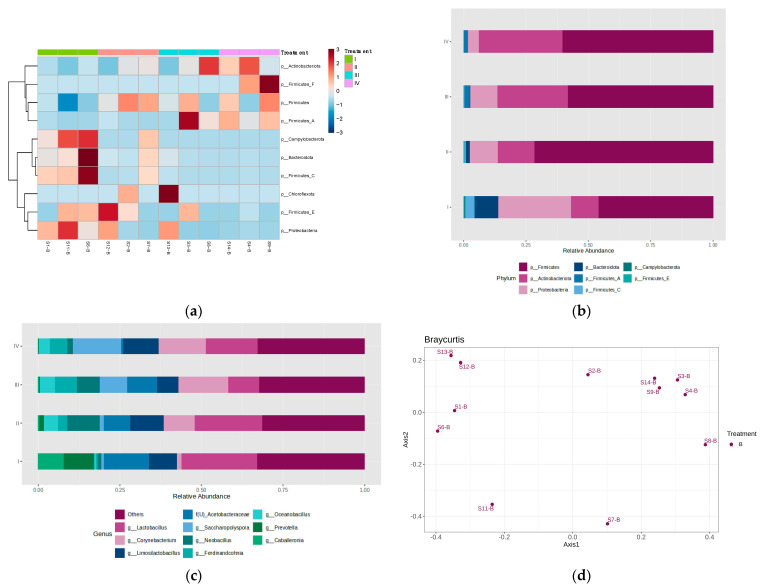
(**a**) Bacterial phyla heatmap and bar plots of bacterial (**b**) phyla and (**c**) top ten genera, depicting the abundance and clustering in the samples taken from Stages I, II, III, and IV of in-vessel composting. A beta diversity analysis (**d**) showcases similarity of bacterial composition of samples.

**Figure 3:**
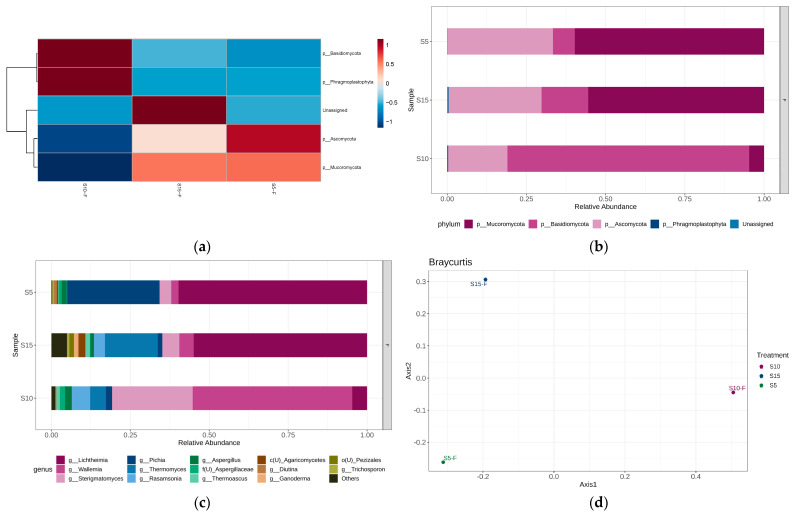
(**a**) Fungal phyla heatmap and bar plots of fungal (**b**) phyla, and (**c**) top ten genera, depicting the abundance and clustering in the samples taken from Stages I, II, III, and IV of in-vessel composting amongst the replicate samples from Stages IV of IVC by phylum. A beta diversity analysis (**d**) showcases the microbial composition similarity of samples.

Additionally, Figure 2 and Figure 3 show the phyla heatmaps and bar plots of the relative abundance of bacterial and fungal phyla and genera in each stage of IVC. 

#### 3.2.1. Metagenomic Bacterial Profile: Notable Bacterial ASVs

A number of unique bacterial ASVs were found at each stage of the IVC composting process. Among those bacterial ASVs were ASVs encoding for *Lactobacillus* spp. (*L. delbrueckii*) [3]; the bacterial class *Bacilli*; a *Bacillus* spp. (*B. smithii*); and an *Acinetobacter* spp. (*A. variabilis*). These ASVs were unique to Stages I, II, III, and IV of the IVC process, respectively.

*Lactobacilli* are anaerobic bacteria with the ability to survive acidic conditions commonly associated with fermentation and are found in abundance in the earlier stages of composting, playing a key role in the metabolic activity of compost microbiota and contributing to CH_4_ and H_2_S emissions during aerobic composting [3,6]. Looking more specifically, *L. delbrueckii* is a functional *Lactobacillus* that has been positively correlated with the polysaccharide and protein contents as well as C/N ratios in compost [3]. John et al. [20] observed *L. delbrueckii* to be a key producer of lactic acid from the fermentation of agricultural wastes. This suggests that the presence of *L. delbrueckii* as a unique ASV of Stage I was not out of place and that *L. delbrueckii* may have contributed to the initial digestion and utilization of available sugars within feedstock. 

*Bacillus* spp. are thermophilic bacteria that dominate the hotter stages of composting—where temperatures increase to around 50 °C—in terms of abundance, contributing to the hydrolytic activity and decomposition of substrates during that stage [3,21]. *Bacillus* spp. *B. smithii* is therefore unsurprisingly similarly characterized as a thermophilic bacterium. Its other properties as determined by De Bartolomeo et al. [22] include positive catalase activity, starch hydrolysis, urase production, and acidification of glucose, arabinose, xylose, and mannitol sugars to different degrees. In composting, Charbonneau et al. [21] isolated *B. smithii* as one of ten thermophilic bacteria from manure compost, observing that it exhibits lipolytic activity in olive oil. Hence, it can be hypothesized that the presence of *B. smithii* in Stage III of the IVC composting process is involved in the digestion of feedstock sugars and fats during the thermophilic stage.

Finally, an ASV for an *Acinetobacter* spp., *A. variabilis*, was found to be unique to Stage IV of the IVC process. Meng et al. [23] and Milanović et al. [24] observed *Acinetobacter* as one of the dominant genera in the initial compost matrix during the mesophilic stages of the composting process, which is displaced by other bacterial species in later stages, suggesting that the presence of *A. variabilis* in Stage IV may be very small. *A. variabilis* has been identified by Ash et al. [25] as a potent source of alkaline proteases, which indicates a possible role of *A. variabilis* in protein degradation of whatever remaining substrates are found during the maturation stage. However, as the pH of the IVC compost output averages pH 6.1 (Table 3), thus being slightly acidic, the hydrolytic activity of *A. variabilis* may be minimal.

#### 3.2.2. Metagenomic Bacterial Profile: Bacterial Abundance by Phyla Across Stages

**Figure 4:**
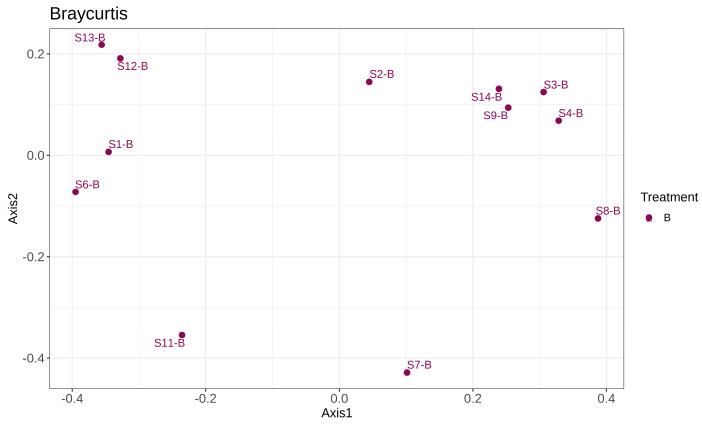
The principle coordinate analysis (PCoA) plot of similarity of samples in microbial composition, analyzed based on the Bray–Curtis dissimilarity matrix.

The beta diversity of individual bacterial samples was elucidated on a principle coordinate analysis (PCoA) graph based on the Bray–Curtis dissimilarity matrix (Figure 4). Samples with greater proximity to each other on the graph share a more similar microbial composition. Replicate samples were found to have dissimilar microbial compositions despite being sampled at the same stage of IVC composting. Two replicates from Stage I of IVC (S1-B, S6-B) were more similar in composition than the third replicate (S11-B), while Stage II (S2-B, S7-B, S12-B) and Stage III (S3-B, S8-B, S13-B) replicates were greatly dissimilar to each other. Stage IV replicates (S4-B, S9-B, S14-B) were found to be most similar, with S9-B and S14-B replicates being more alike but still fairly similar to S4-B. Overall, it can be described that the replicates were similar in composition at Stage I before diverging and becoming dissimilar in Stages II and III before becoming similar in Stage IV.

This dissimilarity in Stages II and III may be because the replicates were sampled from separate composting cycles that occurred months away from each other. At Stage I, the same initial feedstock of sawdust and partially fermented compost was added, so the replicates’ microbial compositions were not dissimilar as the composting process had yet to begin. At Stages II and III, active composting occurs. Graca et al. [2] demonstrated that during this process of composting, the increasing microbial activity leads to an increasing temperature and a significant increase microbial diversity. As the IVC unit used for this study was without an external heat source, there was no strict control on the temperature or humidity of the process. This was because the main intention of this IVC process was to be economical while still providing a high-quality output. However, in allowing the compost to naturally develop and mature, this may have resulted in the variability of the microbial content seen in Stage II and III replicates. At Stage IV of the IVC process, the microbial compositions of replicates were similar, despite being vastly dissimilar at Stage III. One possible reasoning for this change in microbial composition is that after composting, once allowed to settle to room temperature, the microbial populations that thrived at a higher temperature were unable to function at the lower temperature, leaving behind a microbial composition similar to other replicates. The similarity of Stage IV replicates, despite earlier dissimilarity, also proves that the IVC compost design is able to produce output that is of consistent quality and microbial content.

Through an overview of the heatmap in Figure 2a, bacterial ASVs from the phyla *Proteobacteria*, *Campylobacterota*, *Bacteroidota*, *Acintobacteriota*, and *Firmicutes* were found in varying abundance throughout the four stages, with certain subcategories of *Firmicutes* being more abundant at some stages than others. This variety of bacterial phyla abundance is similar to that found by Zhong et al. [26], Partenan et al. [27], and Meng et al. [23] with slight differences—Zhong et al. [26] noted an abundance of *Chloroflexota* in their research on open-air composting (OAC) of dairy manure, and Partenan et al. [26] found bacteria belonging to the *Deinococcus-Thermus* phylum during the stages of aerated tunnel composting of municipal biowaste mixed with wood chips. Meanwhile, in the composting of cow manure and corn straw, Meng et al. [23] detected an abundance of *Chloroflexota*, *Gemmatimonadetes*, and *Planctomycetes*.

These differences in raw materials and composting methods across the studies may explain these small differences in the types of bacterial phyla found during composting, as these are factors known to bring a significant change to the microbial composition of compost [23,26,27].

The total taxa abundances of all four stages show that bacterial ASVs belonging to the *Firmicutes* phyla are greatly abundant throughout the IVC process, with a percentage of 58% abundance that excludes the abundance subcategories of *Firmicutes A* and *C*, which would bring that percentage up to over 60%. *Firmicutes* is known to play a considerable role during the thermophilic stage of composting, as bacteria in this phylum are characterized by their effective carbohydrate metabolism and tolerance for unfavorable conditions [23].

Closer analysis the heatmap (Figure 2a) shows that *Bacteroidota* (i.e., *Bacteroidetes*), *Firmicutes C*, and *Campylobacterota* phyla were found to be particularly abundant during Stage I of IVC, followed by a moderate abundance of *Proteobacteria* and *Firmicutes E*. At Stage II of IVC, bacteria under the phyla *Firmicutes E* and *Firmicutes* show abundance in more than one sample from Stage II, while Stage III exhibits sparse clustering of bacterial phyla across samples, with the phylum *Firmicutes A* being of particular interest. At Stage IV of IVC, the bacterial abundance of ASVs in phyla *Actinobacteriota*, *Firmicutes F*, *Firmicutes,* and *Firmicutes A* can be clearly seen.

The bar chart in Figure 2b further clarifies the changes in bacterial phyla from one stage to the next, denoting a clear decrease in abundance of *Bacteroidota* and *Campylobacterota* as the composting process progressed from Stage I of IVC, and a notable continued increase in *Actinobacteriota* from Stage I to Stages II, III, and IV. Bacteria from the *Bacteroidetes* and *Actinobacteriota* phyla are known as key contributors to the maturation process of composting, while *Campylobacterota* plays a role in the regulation of sulfur and nitrogen cycles in karst coastal and limestone cave waters, performing sulfur oxidation and nitrate reduction [28,29]. *Campylobacterota* bacteria are also notably mesophilic, with an optimal temperature preference of 24–29 °C, providing an explanation for the decrease in its abundance as the observed IVC composting progresses to its thermophilic stages [30]. 

Figure 2c highlights the top 10 genera present in the stages of IVC, showing the changing trends in relative abundance. An increased abundance of bacteria of the genera *Prevotella* and *Caballeronia* and a decreased abundance of genera *Neobacillus*, *Oceanobacillus*, *Ferdinandchonia*, *Saccharopolyspora,* and *Corynebacterium* were initially observed in Stage I. After Stage I, genus *Prevotella* and genus *Caballeronia* sharply decreased in abundance in Stage II and continued to decrease in Stages III and IV. Bacteria from the genus *Neobacillus* were observed to have the greatest abundance in Stage II of IVC, before decreasing in later stages, while bacteria of genus *Ferdinandcohnia* peaked in abundance in Stage III, decreasing in Stage IV. Meanwhile, bacteria of genus *Saccharopolyspora* and *Corynebacterium* showed a distinctive, continued increase in abundance after Stage I, with genus *Saccharopolyspora* reaching its greatest abundance in Stage IV of the IVC process and genus *Corynebacterium* observed to have similar relative abundances in Stages III and IV. Genus *Oceanobacillus,* on the other hand, was seen to have consistent abundance in Stages II, IIIs and IV. 

Additionally, the genus *Lactobacillus* has been found to be present throughout the four IVC composting stages despite previous studies noting a decrease and disappearance of *Lactobacillus* after the initial stages of composting [6]. This discrepancy may be due to the different initial feedstock used, which is a known factor that affects the microbial composition of compost output [2]. The presence of *Lactobacillus* throughout the IVC composting process may explain the lower organic matter content (~60%) (Table 3) and thus higher maturity level of the final compost output, as the continued presence of *Lactobacillus* would allow for the further digestion of sugar substrates that are made available through decomposition. 

#### 3.2.3. Metagenomic Fungal Profile: Notable Fungal ASVs

Only compost samples from Stage IV of the IVC composting process—compost right before packaging—were sampled for assessment of the fungal profile and composition. From the three result replicates, 30 common ASVs of fungal clusters were found with notable ASVs coding for the *Trichosporon* genus, a *Pichia* spp. (*P. deserticola*) and two *Lichtheimia* spp.

*Trichosporon* spp. has been previously detected during the composting of sewage sludge and sawdust by Wang et al. [31], who observed a positive correlation between *Trichosporon* and compost moisture. The same study also noted the ability of *Trichosporon* fungi to utilize a wide variety of substrates and digest lignocellulose into lipids, suggesting the presence of *Trichosporon* spp. to be advantageous in the decomposition of lignocellulosic agro-wastes [31]. However, the *Trichosporon* genus is generally considered to contain opportunistic pathogens, which pose a concern to human health [31,32]. The abundance of *Trichosporon* fungi has been seen to decrease after the thermophilic stage of composting, minimizing the pathogenic concern of the final product [32]. Yet, notable detection of *Trichosporon* in this study was seen at Stage IV of the IVC composting process, past the thermophilic stage, and thus is notable. Even so, the compost output tested negative for the presence of pathogens (Table 3), which may suggest that the *Trichosporon* spp. present are non-pathogenic or in low abundance, meaning they do not affect human health.

*Pichia* spp. are well-known thermotolerant fungi species that play prominent roles in fermentation within the food industry, involved in winemaking, alcohol, cocoa bean fermentation, and many more [33,34]. Since it is able to produce considerable amounts of alcohol, esters, and organic acids during fermentation, it can be extrapolated that *Pichia* spp. would contribute to the content of humic acid (Table 3) measured from the IVC compost output and to the fermentation and curing phase of composting in general [33].

The specific *Pichia* spp. detected in Stage IV of the IVC process was *P. deserticola*. Originally isolated from the necrotic stems of Opuntia cacti tissues, *P. deserticola* was determined to be a fungal species without the ability to ferment glucose, instead exhibiting the ability to assimilate certain nitrogen compounds such as ethylamine, lysine, and ammonium sulfate [35]. It was also one of the many fungal species observed during the fermentation of cocoa beans, contributing to the synthesis of aromatic compounds [34].

Finally, two *Lichtheimia* spp. were also detected during Stage IV of the IVC process. The *Lichtheimia* fungal genus can produce polygalacturonase and xylanase, and it is not unheard of to find this genus in composts where they might possibly be contributing to the breakdown of agricultural substrates [32]. The thermotolerant *Lichtheimia* sp. *L. ramosa* has been found to be present in the composting of compressed sugar industry wastes and characterized to have high ß-galactosidase production, aiding in the breakdown of lactose sugars [32,36]. However, the *Lichtheimia* spp. observed in this study were *L. ornata* and *L. corymbifera*. Volford et al. [36] isolated *L. corymbifera* from a soil sample and noted high ß-galactosidase activity of the species on X-gal, showing potential as a source for producing ß-galactosidase from the utilization of agro-industrial feedstocks. The effects and contributions of these two *Lichtheimia* spp. to composting are not well-elucidated and would require further research.

#### 3.2.4. Metagenomic Bacterial Profile: Fungal Abundance by Phyla During Stage IV

**Figure 5:**
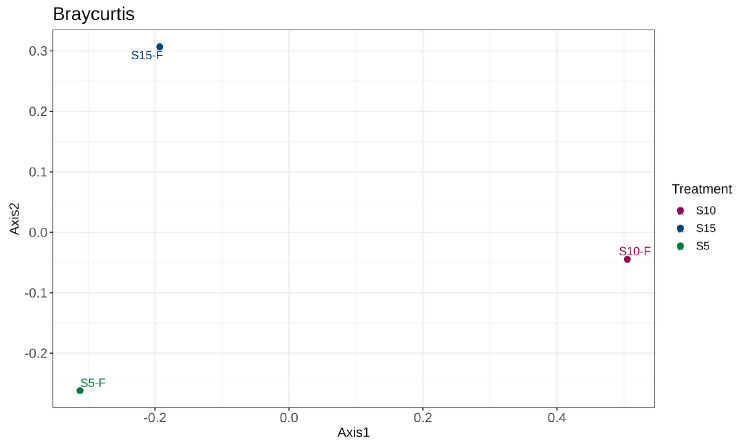
The principle coordinate analysis (PCoA) plot of similarity of samples in fungal composition of Stage IV sample replicates, analyzed based on the Bray–Curtis dissimilarity matrix.

The beta diversities of sample replicates at Stage IV of the IVC process were found to be greatly dissimilar from each other (Figure 5). Similar to the divergence in bacterial composition of other stages of the IVC process, this dissimilarity seen in sample replicates S5-F, S10-F, and S15-F may be due to lack of strict temperature and humidity control of the separate cycles of IVC composting. Despite the overall dissimilarity, the relative fungal abundance during Stage IV of IVC, as visualized in Figure 3b,c at the phylum level and genus level, respectively, does show overlap in fungal phyla and genera present in the samples. 

As a whole, the fungal phylum *Basidiomycota* was found to be the most abundant, followed by *Mucoromycota* (i.e., *Mucoromycetes*) and *Ascomycota*, present in all three replicates. However, Figure 3a,b showcases the spread of taxa abundances across the three sample replicates, and it should be noted that fungal ASVs from the *Basidiomycota* phylum were greatly concentrated in the second replicate sample, S-10F. Therefore, we considered the overall abundance of *Basidiomycota* as secondary to that of fungi phyla of *Mucoromycota* and *Ascomycota*.

At the genus level, a greater overall abundance of fungi from the *Lichtheimia* genus was seen in replicates S-5F and S-15F. Replicate S-10F showed a marked difference in composition, with greater-abundance fungi of the *Wallemia* and *Sterigmatomyces* genera in comparison to the other two replicates, which had a much lower abundance of these two genera. Of the replicates, replicate S-15F had the greatest variety of fungal genera and a higher abundance of *Thermomyces,* whereas replicate S-5F showcased the highest abundance of fungi of the genus *Pichia*.

On a phylum level, other studies have found similar results that corroborate those observed here. Meng et al. [23] and Langarica-Fuentes et al. [13] noted an abundance of *Ascomycota* followed by *Basidiomycota* with Langarica-Fuentes et al. [13], also noting an abundance of the *Mucoromycota* sub-phylum. Like with bacterial composition, this can be explained by the differences in the in-feed material of compost, process methods, and conditions for composting across the two studies.

As previously mentioned, *Pichia* spp. are thermotolerant fungi involved in fermentation—particularly that of food and wine—with the ability to produce alcohol, esters, and organic acids that contribute to the overall compost quality and composition [33,34]. *Thermomyces* spp., meanwhile, are known for their thermophilicity and thermotolerance along with their ability to produce thermostable or resistant enzymes that contribute to the composting process [37]. Finally, *Lichtheimia* spp., with their production of polygalacturonase and xylanase, contribute to the breakdown of agricultural in-feed [32].

## 4. Conclusions

A pilot aerobic in-vessel composting system was successfully developed, producing compost with a 50% yield within a short retention time of 7–10 days. With this optimized feedstock, the compost produced by the IVC method was shown to be of a higher quality based on the maturity, pH, NPK, organic matters, and macronutrients contents. This study also provides novel insights into the bacterial and fungal composition present in compost of the unique optimized feedstock. The metagenomic analysis presented the bacterial and fungal compositions of the compost with the differences in species composition as the result of the different initial substrates utilized. The top five bacterial amplicon sequence variants (ASVs) found in all stages were *Lactobacillus* (Genus)*, Acetobacteraceae* (Family)*, Corynebacterium* (Genus)*, Saccharopolyspora rectivirgula* (Species)*,* and *Neobacillus* (Genus); and the top five shared fungal clusters were *Trichosporon* (Genus)*, Sterigmatomyces* (Genus)*, Pichia deserticola* (Species)*, Lichtheimia ornata* (Species)*,* and *Lichtheimia corymbifera* (Species). Bacterial ASVs from the phyla *Proteobacteria*, *Campylobacterota*, *Bacteroidota*, *Acintobacteriota*, and *Firmicutes* were found in varying abundance throughout the four stages, with certain subcategories of Firmicutes being more abundant at some stages than others. In addition, ASVs encoding a *Lactobacillus* spp., the bacterial class *Bacilli*, a *Bacillus* spp., and an *Acinetobacter* spp. were identified to be the unique bacterial ASVs in each stage, and the *Trichosporon* genus, a *Pichia* spp. (*P. deserticola*), and two *Lichtheimia* spp. were found to be the notable fungal ASVs.

## Figures and Tables

**Table 1:** Characterization methods.

Parameter	Analysis Method	Analysis Unit
Moisture Content (%)	AOAC931.04, Oven method	Conducted in lab
pH at 25 °C	AOAC945.10	Conducted on-site
Temperature	Temperature probe, at the bottom, middle, and upper layers of the composting system	Conducted on-site
Total Organic Carbon, C (%)	MS 417: Part 8: 1997	Outsourced to BVAQ, Petaling Jaya, Malaysia
Total Nitrogen, N (%)	MS 417: Part 3: 1994 and AOAC991.20	
Bulk Density (kg/m^3^)	ASTM D1895B	
Humic Acid	HG/T 3278-2011	
Nitrate Nitrogen, NO_3_-N (mg/kg)	APHA 418E	Outsourced to BVAQ, Petaling Jaya, Malaysia
Conductivity	APHA 2510B	
Phosphorus, P_2_O_5_	MS 417: Part 4: 1994	
Potassium, K_2_O	MS 417: 1994 and 1997	
Magnesium, MgO	MS 417: 1994 and 1997	
Calcium, CaO	US EPA 6010B (1996) (ICP-OES) and AOAC 975.03	
Sulfur, S		
Iron, Fe		
Boron, B		
Zinc, Zn		
Manganese, Mn		
Copper, Cu		
Sodium, Na		
Aluminum, Al		
Cobalt, Co		
Nickel, Ni		
Molybdenum, Mo		

**Table 2:** Three (3) scenarios of substrate formulation and their resultant output from IVC.

SCENARIO	INPUT (Tons/Month)						OUTPUT		
	Other [Dataset]	SCR (Dry)	SCR (Wet)	Saw- Dust	Partially Fermented Compost ^1^	Total	Amount Produced ^2^ (Tons/Month)	Moisture Content (%)	Lead Time (Days)
A	80	62.7	130	40	0	312.7	149	32	7
	0	60	110	80	0	250.0	86	28	10
B	0	0	0	60	180	240.0	123	25	7
	80	62.7	130	40	0	312.7	149	32	7
	0	60	110	80	0	250.0	86	28	10
	0	0	0	60	180	240.0	123	25	7
C	80	62.7	130	40	0	312.7	149	32	7
	0	60	110	80	0	250.0	86	28	10

^1^ Partially fermented compost refers to 1–2-week-old compost produced by the OAC method using a mixture of SCR and sawdust as feedstock. ^2^ Amount of output was calculated following Equations (3)–(5).

**Table 3:** Assessment of IVC output from optimized feedstock (Scenario C) based on key parameters of compost quality. Three (3) scenarios of substrate formulation and their resultant output from IVC.

Key Parameters	IVC Compost			Average
	September 2020	October 2020	January 2021	
NPK (%)	±7	±5	±8	±6
Moisture Content, (%)	29.6	25.1	28.9	27.9
pH	5.2	5.31	7.8	6.1
Organic Matter (%)	58.5	62.6	84.1	68.4
Total Organic Carbon, C (%)	34.0	36.3	48.8	39.7
C/N Ratio	18:1	20:1	18:1	18.7:1
Conductivity (µS/cm)	6620	5560	2880	5020
Humic Acid (%)	26.9	86.8	n/a	56.85
Bulk Density (kg/m^3^)	673	554	439	555.3
Nitrate Nitrogen, NO_3_-N (mg/kg)	5009	89.2	2.3	1700.2
Solvita Index	3	3	7	4.3
Particle Size	<3.0 mm			
Appearance	Loose, friable, powder form			
Color	Dark brown			
Presence of Pathogens	Absent			

**Table 4:** Macronutrient content of IVC compost output.

Macronutrients	Content (% *w*/*w*)			Average (% *w*/*w*)
	September 2020	October 2020	January 2021	
Total Nitrogen, N	1.92	1.83	3.8	2.52
Phosphorus, P_2_O_5_	2.02	1.93	2.4	2.12
Potassium, K_2_O	3.01	1.54	2.4	2.32
Magnesium, MgO	2.06	1.06	0.6	1.24
Calcium, CaO	3.30	1.03	2.3	2.21
Sulfur, S	0.37	0.12	ND (<0.1)	0.25
Iron, Fe	9674	2420	7000	6365
Boron, B	7.6	10.2	173.8	63.87
Zinc, Zn	55.5	22.2	91.3	57.83
Manganese, Mn	398	131	107.5	212.17
Copper, Cu	24.5	11.2	52.4	29.37
Sodium, Na	6040	332	7000	4457
Aluminum, Al	685	2617	3000	6302
Cobalt, Co	2.4	1.02	ND (<0.1)	1.71
Nickel, Ni	17.1	8.3	0.69	8.70
Molybdenum, Mo	<0.1	0.15	ND (<0.1)	<0.125

**Table 5:** Top 5 bacterial ASVs unique to each IVC stage: Stage I, Stage II, Stage III, and Stage 4.

Stage I	Stage II	Stage III	Stage IV
*Lactobacillus delbrueckii* * (Species)	*Bacilli* * (Class)	*Ferdinandcohnia* (Genus)	*Haloplasma contractile* (Species)
*Enterobacteriaceae* (Family)	*Clostridia* (Class)	*Lihuaxuella thermophila* (Species)	*Acinetobacter variabilis* (Species)
*Salmonella* (Genus)	*Siminovitchia* (Genus)	*Bacillus O smithii* (Species)	*Acinetobacter* (Genus)
*Megasphaera elsdenii* (Species)	*Paenibacillaceae* (Family)	*Lachnospiraceae* (Family)	*Actinomycetia* (Class)
*Acinetobacter* (Genus)	*Aeriscardovia aeriphila* (Species)	*UBA11021 sp013664685* (Species)	*Pseudoxanthomonas* (Genus)

* Unique ASVs conferred to the same class were counted as a single unique ASV, and the following unique ASV of a high OTU count was included.

## Data Availability

Third-party data. Restrictions apply to the availability of these data. Data were obtained from University of Nottingham Malaysia and are available from the authors with the permission of the University of Nottingham Malaysia.
